# Characterization and phylogenetic analysis of the complete mitochondrial genome sequence of *Diospyros oleifera*, the first representative from the family Ebenaceae

**DOI:** 10.1016/j.heliyon.2022.e09870

**Published:** 2022-07-06

**Authors:** Yang Xu, Yi Dong, Wenqiang Cheng, Kaiyun Wu, Haidong Gao, Lei Liu, Lei Xu, Bangchu Gong

**Affiliations:** a Research Institute of Subtropical Forestry, Chinese Academy of Forestry, Hangzhou, 311400, China; b Genepioneer Biotechnologies Co. Ltd, Nanjing, 210023, China

**Keywords:** *Diospyros oleifera*, Mitochondrial genome, Phylogenetic analysis

## Abstract

Plant mitochondrial genomes are a valuable source of genetic information for a better understanding of phylogenetic relationships. However, no mitochondrial genome of any species in Ebenaceae has been reported. In this study, we reported the first mitochondrial genome of an Ebenaceae model plant *Diospyros oleifera*. The mitogenome was 493,958 bp in length, contained 39 protein-coding genes, 27 transfer RNA genes, and 3 ribosomal RNA genes. The rps2 and rps11 genes were missing in the *D. oleifera* mt genome, while the rps10 gene was identified. The length of the repetitive sequence in the *D. oleifera* mt genome was 31 kb, accounting for 6.33%. A clear bias in RNA-editing sites were found in the *D. oleifera* mt genome. We also detected 28 chloroplast-derived fragments significantly associated with *D. oleifera* mt genes, indicating intracellular tRNA genes transferred frequently from chloroplasts to mitochondria in *D. oleifera*. Phylogenetic analysis based on the mt genomes of *D. oleifera* and 27 other taxa reflected the exact evolutionary and taxonomic status of *D. oleifera*. Ka/Ks analysis revealed that 95.16% of the protein-coding genes in the *D. oleifera* mt genome had undergone negative selections. But, the rearrangement of mitochondrial genes has been widely occur among *D. oleifera* and these observed species. These results will lay the foundation for identifying further evolutionary relationships within Ebenaceae.

## 1 Introduction

Mitochondria are the main organelle involved in energy metabolism in plants [1, 2]. They supply ATP via oxidative phosphorylation for metabolism, cell differentiation, apoptosis, cell growth, and cell division and are abundant in energy-consuming tissues involved in essential biological functions [1, 2, 3, 4]. Therefore, mitochondria play an important role in plant productivity and development [2, 5, 6]. According to endosymbiotic theory, plant mitochondria are believed to have descended from free-living bacteria-independent microorganisms, which explains the presence of their genomes [5, 7].

During evolution, the plant mitochondrial (mt) genome underwent dramatic changes in, for example, the gene order, genome structure, and migration of sequences from other organelles [5, 7, 8, 9]. Thus, plants have about 100–10,000 times larger and more structurally complex mitochondrial (mt) genomes than animals [10, 11, 12]. The mt genomes of plants demonstrate significant genome size variation, from 66 kb [13] to 11.3 Mb [14]; the number of protein-coding genes varies from 14 to 67 [15]; and the number of tRNA genes varies from 3 to 27 [9]. There are variations in mitochondrial genomes not only between plant species but also within the same species [9, 12, 16, 17], in stark contrast to the conserved structure of plant chloroplast genomes [16, 17, 18]. Thus, mt genomes have been used as a valuable source of genetic information and for investigation of essential cellular processes in many phylogenetic studies [18, 19, 20, 21].

While, these characteristics of plant mt genomes (bigger size, more structural complexity, and low conservation across species) make plant mitochondrial genome assembly difficult [1, 8, 10]. To date, more than 5000 plant chloroplast genomes have been sequenced, but only about 400 mt genome sequences are available (www.ncbi.nlm.nih.gov/genome/organelle/,11/11/2021). In addition, sequenced plants largely differ in their classification, and only three complete mitochondrial genomes of species from the order Ericales have been identified.

*Diospyros* L., from the Ebenaceae family, is a plant genus that includes over 500 species widely distributed across tropical and subtropical regions [22] and that is one of the largest angiosperm genera [23]. Among these species, *Diospyros oleifera* and *Diospyros kaki* have been cultivated as an important fruit crop in China, Korea, Japan for centuries, due to its edible fruit is rich in vitamins, sugars, nutrients and antioxidants vital for optimum health with various medicinal and chemical [24, 25]. Morphological, molecular, and genomic studies have shown that *D. oleifera* can be used as a model plant [24, 26]. Chloroplast genome sequencing has been performed in 15 species of *Diospyros* [26, 27], and nuclear genome sequencing has been performed in *D. oleifera* [23, 28] and *Diospyros lotus* [29, 30]. However, to date, no mt genome of any species in Ebenaceae has been reported.

Fortunately, advancements in long read sequencing, such as PacBio and Oxford Nanopore, have made organelle genome sequencing easier and faster. Therefore, in this study, we constructed the complete mt genome of *D. oleifera* based on PacBio and Illumina data, performed a phylogenetic analysis, and compared the complete mt genomes of *D. oleifera* and related genera. These results will help better understand the features of the *D. oleifera* mitochondrial genome and lay the foundation for identifying further evolutionary relationships within Ebenaceae.

## 2 Materials and methods

### 2.1 Samples and mitogenome sequencing

Due to the advancement of sequence technology, long reads, used for *de novo* assembly of organelle genomes without the need for organelle DNA isolation, could be easily generated from high throughput sequencing. The well-established methodology is quite efficient and well accepted in the scientific community [1, 31, 32, 33, 34, 35, 36, 37, 38, 39, 40, 41, 42].

In this study, Mature leaves of *D. oleifera* (at latitude 34.27569 and longitude 107.75079) were used to isolate total DNA following the protocol for the Illumina HiSeq 2500 platform (Illumina, San Diego, CA, USA) and the SMRTbell Libraries protocol for PacBio data (Pacific Biosciences, Menlo Park, CA, USA). All these whole-genome Illumina HiSeq and PacBio sequencing data were deposited in the NCBI GenBank (accession no. PRJNA562043) and the Persimmon Genome Website (http://www.kakiwi.zju.edu.cn/cgi-bin/persimmon/about_genome.cgi). Sequencing reads of the mitochondria were filtered and extracted from these WGD sequencing data of *D. oleifera*. Raw data of second-generation sequencing were filtered using fastp version 0.20.0 software (https://github.com/OpenGene/fastp) [43]. The three-generation sequencing data of mitochondrial reads were error-corrected, trimmed, and de-novo-assembled using a Canu assembler (version 1.5) with default parameters [44]. Then, the contig sequence was obtained. The gene databases of plant mitochondria (the mitochondrial gene sequences of species published on the NCBI) were compared using blast v2.6 (https://blast.ncbi.nlm.nih.gov/Blast.cgi), and contigs that matched with the mitochondrial gene as the seed sequence were selected. The original data were used to extend and circularize the contigs to obtain the ring-dominant structure (or secondary ring), and then, the assembly was polished using NextPolish 1.3.1 (https://github.com/Nextomics/NextPolish) [45]. The assembly results were calibrated using second- and third-generation data, and the parameters were set as rerun = 3 and -max_depth = 100. Then, the final assembly results were obtained.

### 2.2 Genome annotation

The assembled *D. oleifera* mt genome was annotated using the GeSeq tool [46]. To confirm the annotated results, the assembled *D. oleifera* mt genome was also BLAST-searched against protein-coding genes and ribosomal RNA (rRNA) genes of available plant mt genomes at the NCBI. Then, the sequence coordinates of the identified protein-coding genes (PCGs) were manually verified for start and stop codons. The annotations of transfer RNA (tRNA) genes were also confirmed by tRNAscan-SE [47]. ViennarNA-2.4.14 [48] was used to visualize the secondary structure of tRNA. The physical circular map was drawn using the Organellar Genome DRAW (OGDraw) v1.2 program [49]. The final annotated mt genome sequences of *D. oleifera* have been deposited in the NCBI GenBank (accession no. MW970112).

Strand asymmetry was calculated according to the formulas: AT-skew = [A − T]/[A + T] and GCskew = [G − C]/[G + C] [50]. The possible RNA-editing sites in the PCGs of *D. oleifera* were predicted using the online predictive RNA editor for plant mitochondrial genes (PREP-Mt) [51] suite of servers (http://prep.unl.edu/). The codon frequencies were calculated using the Codon Usage tool in the Sequence Manipulation Suite (bioinformatics.org/sms2/codon_usage.html) [52]. The relative synonymous codon usage (RSCU [53]) was calculated using the CAI Python package of Lee [54].

### 2.3 Analysis of repeated sequences

Three kinds of repeats (simple sequence, tandem, and dispersed) were detected in the *D. oleifera* mitochondrial genome. The MIcroSAtellite (MISA) identification tool Perl script was used to detect simple sequence repeats [55]. The repeats of mono-, di-, tri-, tetra-, penta-, and hexanucleotide bases with 12, 6, 4, 3, 3, and 3 repeat numbers, respectively, were identified. Tandem repeats (>6 bp repeat units) were detected using Tandem Repeats Finder v4.09 software (http://tandem.bu.edu/trf/trf.submit.options.html) [56] with default parameters (matching probability of 80 and indel probability of 10). Direct and inverted repeats were detected using the vmatch (v2.3.0) Perl script with the minimal repeat size set to 30 bp.

### 2.4 Chloroplast-to-mitochondrion-DNA transformation

The *D. oleifera* cp genome (NC_030787.1) was downloaded from the NCBI Organelle Genome Resources Database. The protein-coding and tRNA genes, which were transferred from chloroplasts to mitochondria, were identified using Blastn software with the following screening criteria: matching rate ≥70%, E-value ≤ 1e − 10, and length ≥30 bp.

### 2.5 Phylogenetic tree construction and Ka/Ks analysis

The *D. oleifera* and Twenty-seven other species with complete or nearly complete mitogenomes were used in phylogenetic analyses, representing twenty families. Two species from Conifers were used as outgroup. All species were listed in Table 1. The mt genomes were downloaded from the NCBI Organelle Genome Resources Database, and the conserved protein-coding genes (*atp1*, *atp4*, *atp6*, *atp8*, *atp9*, *ccmB*, *ccmC*, *ccmFC*, *ccmFN*, *cob*, *cox1*, *cox2*, *cox3*, *matR*, *nad1*, *nad2*, *nad3*, *nad4*, *nad4L*, *nad5*, *nad6*, *nad7*, *nad9*, *rpl5*, *rps12*, *rps13*, *rps3*, and *rps4*) were extracted and aligned using MAFFT v7.402 [57] with default parameters. ModelTest-NG v0.1.3 was used to determine the best-fit model, and a maximum likelihood (ML) tree was generated using RAxMLv8.2.12 with the best-fit substitution model (GTRGAMMA) at 1000 bootstrap replicates [58].

**Table 1:** GenBank accession numbers of mitochondrial genomes for species sampled in this study.

Classification Status		Order	Familiy	Species	Length (bp)	Accession number
Ingroup	Asterids	Apiales	Apiaceae	*Daucus carota#*	281,132	NC_017855
	Asterids	Aquifoliales	Aquifoliaceae	*Ilex pubescens*	517,520	NC_045078
	Asterids	Asterales	Asteraceae	*Chrysanthemum boreale*	211,002	NC_039757
	Asterids	Asterales	Asteraceae	*Helianthus annuus*	300,945	NC_023337
	Asterids	Asterales	Asteraceae	*Lactuca sativa*	363,324	NC_042756
	Asterids	Asterales	Asteraceae	*Lactuca serriola*	363,328	NC_042378
	Asterids	Asterales	Campanulaceae	*Codonopsis lanceolata*	403,704	NC_037949
	Asterids	Asterales	Campanulaceae	*Platycodon grandiflorus*	1,249,593	NC_035958
	Asterids	Ericales	Ebenaceae	*Diospyros oleifera∗#*	493,958	MW970112
	Asterids	Ericales	Ericaceae	*Rhododendron simsii#*	802,707	WJXA01000014
	Asterids	Ericales	Ericaceae	*Vaccinium macrocarpon#*	459,678	NC_023338
	Asterids	Gentianales	Rubiaceae	*Scyphiphora hydrophyllacea*	354,155	MT610041
	Asterids	Lamiales	Lamiaceae	*Salvia miltiorrhiza*	499,236	NC_023209
	Asterids	Lamiales	Lentibulariaceae	*Utricularia reniformis*	857,234	NC_034982
	Asterids	Lamiales	Oleaceae	*Olea europaea*	710,808	MW262896
	Asterids	Lamiales	Phrymaceae	*Mimulus guttatus*	525,671	NC_018041
	Asterids	Solanales	Convolvulaceae	*Ipomoea nil*	265,768	NC_031158
	Asterids	Solanales	Solanaceae	*Capsicum annuum*	511,530	KJ865410
	Asterids	Solanales	Solanaceae	*Nicotiana tabacum*	430,597	NC_006581
	Asterids	Solanales	Solanaceae	*Solanum lycopersicum#*	446,257	NC_035963
	Commelinids	Poales	Poaceae	*Oryza sativa*	637,692	JF281153
	Commelinids	Poales	Poaceae	*Zea mays*	680,603	DQ645539.1
	Rosids	Brassicales	Brassicaceae	*Arabidopsis thaliana*	367,808	NC_037304
	Rosids	Fabales	Fabaceae	*Glycine max*	402,558	NC_020455
	Rosids	Rosales	Rosaceae	*Malus domestica#*	396,947	NC_018554
	Rosids	Vitales	Vitaceae	*Vitis vinifera*	773,279	NC_012119
Outgroup	Conifers	Ginkgoales	Ginkgoaceae	*Ginkgo biloba*	346,544	KM672373
	Conifers	Pinales	Pinaceae	*Pinus taeda*	1,191,054	MF991879

∗Represents the new mitogenome in this study. #Represents these species were used for mitogenome synteny and rearrangements through Mauve software.

The synonymous (Ks) and nonsynonymous (Ka) substitution rates of the protein-coding genes in the *D. oleifera* mt genome were analyzed using the 27 species. In this analysis, KaKs_Calculator (v2.0) [59] with the MLWL model was used to calculate Ka/Ks. Genome synteny and rearrangements among the using six representative species (Table 1) mitogenomes were analyzed using the progressive Mauve algorithm as implemented in Mauve ver. 2.4.0 software [60].

## 3 Results and discussion

### 3.1 Genomic features of the *D. oleifera* mt genome

The plant mitochondrial genome greatly varies in size, from 66 kb in *Viscum scurruloideum* [13] to 11.3 Mb in *Silene conica* [14]. We assembled the complete mt genome of *D. oleifera* in a single circular contig of 493,958 bp (GenBank accession number MW970112). The relatively medium size of the *D. oleifera* mt genome is similar to that of *Vaccinium macrocarpon* (459,678 bp) [3] and some asterids, such as *Solanum lycopersicum* (446,257 bp) [61], *Salvia miltiorrhiza* (499,236 bp) [62], and *Capsicum annuum* (511,530 bp) [63]; smaller than that of *Rhododendron simsii* (802,707 bp) [64] and *Olea europaea* (710,808 bp) [65]; and larger than that of *Daucus carota* (281,132 bp) [66] and *Malus domestica* (396,947 bp) [67].

**Figure 1:**
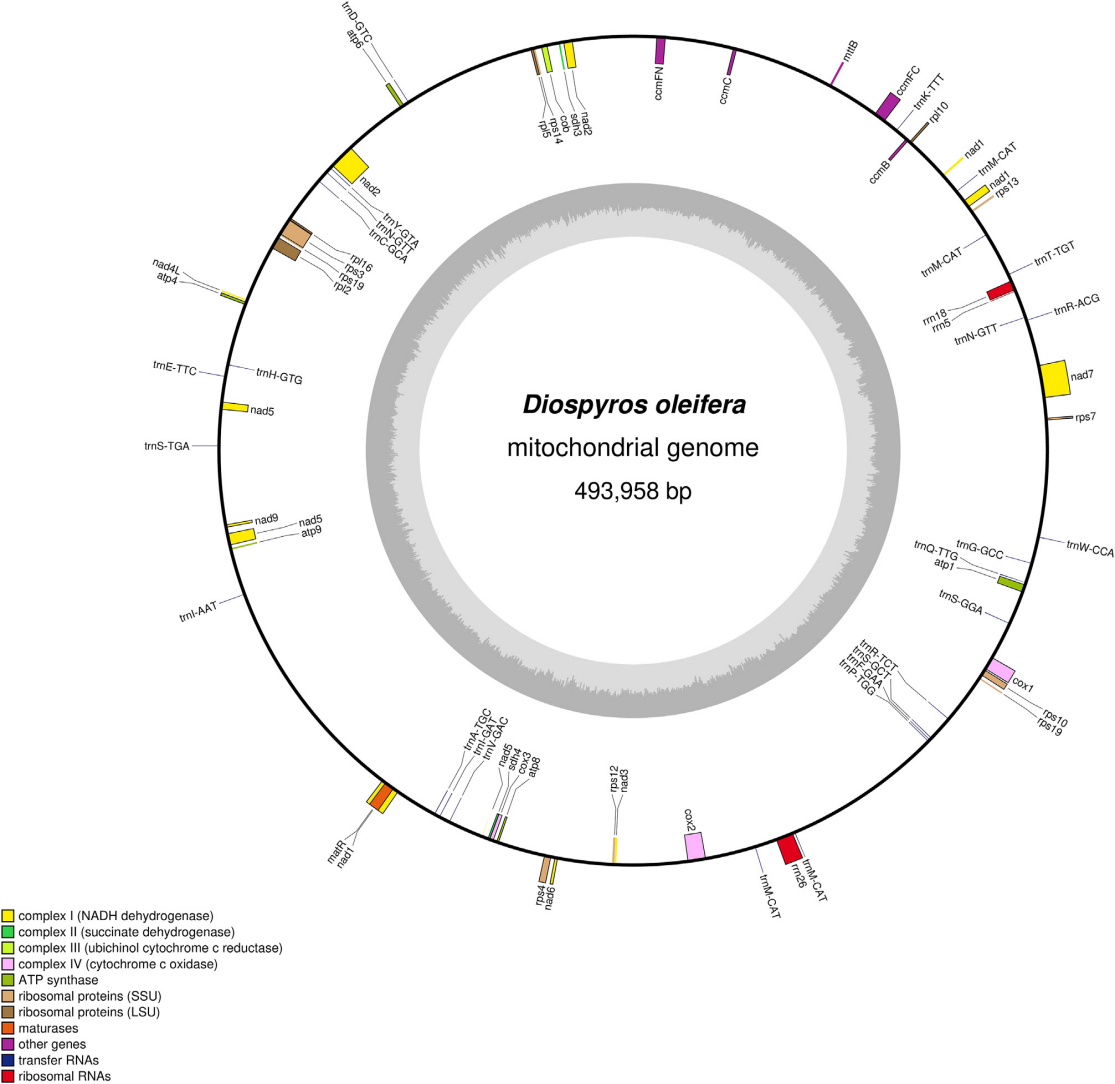
The circular map of *D. oleifera* mt genome. Gene map showing 69 annotated genes of different functional groups.

In the *D. oleifera* mt genome, 69 genes (39 protein-coding genes, 27 tRNA genes, and 3 rRNA genes (*rrn5*, *rrn18*, and *rrn26*)) were annotated. The functional categorization and physical locations of the annotated genes are shown in Figure 1. The 38 different proteins (*rps1*9 has two copies) could be divided into 10 classes (Table 2): ATP synthase (five genes), cytochrome C biogenesis (four genes), ubiquinol cytochrome c reductase (one gene), cytochrome C oxidase (three genes), maturases (one gene), transport membrane protein (one gene), NADH dehydrogenase (nine genes), ribosomal proteins (LSU; four genes), ribosomal proteins (SSU; nine genes), and succinate dehydrogenase (two genes). ATG was used as the starting codon by almost all the protein-coding genes, and the four stop codons (TAA, TGA, TAG, and CGA) had utilization rates of 48.71%, 30.77%, 17.95%, and 2.57%, respectively.

**Table 2:** Gene profile and organization of the *D. oleifera* mt genome.

Group of genes	Gene name	Length	Start codon	Stop codon	Amino acid
ATP synthase	*atp1*	1530	ATG	TGA	510
	*atp4*	579	ATG	TAA	193
	*atp6*	807	ATG	TGA	269
	*atp8*	480	ATG	TAA	160
	*atp9*	285	ATG	TAG	95
Cytohrome c biogenesis	*ccmB*	621	ATG	TGA	207
	*ccmC*	753	ATG	TGA	251
	*ccmFC∗*	1353	ATG	TAA	451
	*ccmFN*	1755	ATG	TGA	585
Ubichinol cytochrome c reductase	*cob*	1182	ATG	TGA	394
Cytochrome c oxidase	*cox1∗*	1584	ACG (ATG)	TAA	528
	*cox2∗∗*	780	ATG	TAA	260
	*cox3*	798	ATG	TGA	266
Maturases	*matR*	1968	ATG	TAG	656
Transport membrance protein	*mttB*	375	ATG	TAG	125
NADH dehydrogenase	*nad1∗∗∗∗*	978	ATG	TAA	326
	*nad2∗∗∗∗*	1467	ATG	TAA	489
	*nad3*	357	ATG	TAA	119
	*nad4∗∗∗*	1488	ATG	TGA	496
	*nad4L*	273	ATG	TAA	91
	*nad5∗∗∗∗*	2013	ATG	TAA	671
	*nad6*	618	ATG	TAA	206
	*nad7∗∗∗∗*	1185	ATG	TAG	395
	*nad9*	588	ATG	TAG	196
Ribosomal proteins (LSU)	*rpl10*	489	ATG	TAA	163
	*rpl16*	435	ND	TAA	145
	*rpl2∗*	1005	ATG	TAA	335
	*rpl5*	564	ATG	TAA	188
Ribosomal proteins (SSU)	*rps10∗*	333	ACG (ATG)	CGA	111
	*rps12*	378	ATG	TGA	126
	*rps13*	351	ATG	TGA	117
	*rps14*	303	ATG	TAG	101
	*rps19(2)*	(231,231)	ATG	TAA	77
	*rps3∗*	1752	ATG	TAG	584
	*rps4*	1326	ATG	TAA	442
	*rps7*	447	ATG	TAA	149
Succinate dehydrogenase	*sdh3*	306	ATG	TGA	102
	*sdh4*	432	ATG	TGA	144
Ribosomal RNAs	*rrn18*	1904			
	*rrn26*	3373			
	*rrn5*	119			
Transfer RNAs	*trnA-TGC∗*	67			
	*trnC-GCA*	71			
	*trnD-GTC*	74			
	*trnE-TTC*	72			
	*trnF-GAA*	74			
	*trnG-GCC*	72			
	*trnH-GTG*	74			
	*trnI-AAT*	69			
	*trnI-GAT∗*	74			
	*trnK-TTT*	73			
	*trnM-CAT(4)*	(73,74,74,77)			
	*trnN-GTT(2)*	(72,72)			
	*trnP-TGG*	75			
	*trnQ-TTG*	72			
	*trnR-ACG*	74			
	*trnR-TCT∗*	72			
	*trnS-GCT*	88			
	*trnS-GGA*	87			
	*trnS-TGA*	87			
	*trnT-TGT∗*	75			
	*trnV-GAC*	72			
	*trnW-CCA*	74			
	*trnY-GTA*	83			

Previous studies have shown that *rps10* is missing in the mt genomes of most plants, such as *Arabidopsis thaliana*, *Brassica napus,* and *Beta vulgaris*, and that its function is replaced by the nuclear gene [9]. However, the *rps10* gene was found in the *D. oleifera* mt genome. The absence of *rps2* and *rps11* genes in the *D. oleifera* mt genome, consistent with *R. simsii* [64] and *V. macrocarpon* [3], supports Adams’ speculation that *rps2* and *rps11* genes were lost in the early evolution of eukaryotic plants [3]. Similar to *Nicotiana tabacum* [68] and *M. luteus* [4], the *D. oleifera* mt genome has no *rps1* gene, whereas *rps1* is present in the *V. macrocarpon* mt genome [3] and two copies of *rps1* are present in the *R. simsii* mt genome [64].

**Figure 2:**
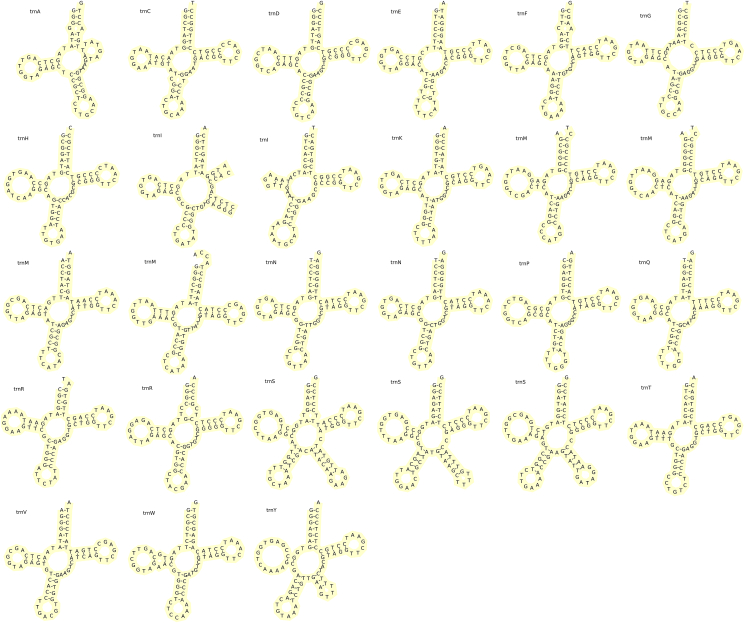
Secondary structures of tRNAs of *D. oleifera*. Each region of tRNA is named as follows [69, 70]: Amino acid accepting stem, AAS (upper arm); dihydrouridine stem and loop, DSL (left arm); anticodon stem and loop, ASL (lower arm); thymidine stem and loop, TSL (right arm); variable stem and loop, VSL (between ASL and TSL).

The persimmon mitochondria have 27 tRNAs (23 typical tRNA genes, one more *trnN-GTT* and three more *trnM-CAT*). The average length of these tRNAs is 67–88 bp, with a total length of 1479 bp. The number of tRNAs in the *D. oleifera* mt genome is more than that in other asterids, such as *V. macrocarpon* (18) [3], *R. simsii* (23) [64], *M. luteus* (24) [4], and *N. tabacum* (21) [68]. This may be because some tRNAs in the *D. oleifera* mt genome have multiple copies; for example, *trnN-GTT* has two copies and *trnM-CAT* has four copies. The secondary structures are shown in Figure 2. Following terms for Agris et al. [69, 70], secondary structures of most tRNAs were recovered as ordinal cloverleaf structures, which includes amino acid accepting stem (AAS), dihydrouridine stem and loop (DSL), anticodon stem and loop (ASL), thymidine stem and loop (TSL), furthermore, *trnI-GAT*, *trnS-GCT*, *trnS-GGA*, *trnS-TGA*, *trnY-GTA* were with an addition variable stem and loop (VSL). And, consist with many report [19, 21, 38, 39, 42], G-T (U) matches were also found in mostly tRNA secondary structures in the *D. oleifera* mt genome.

The total gene length added up to 8% of the total mt genome length, with protein-coding regions comprising only 6.5% (32 kb) of the genome length. The gene content of *D. oleifera* is similar to that of the published mt genomes of asterids, especially *Mimulus guttatus* (7.4%) [4] and *Helianthus annuus* (8.5%) [71]. We found 54 genes with no introns, accounting for 78.26% of the total, consistent with the result conclusion that 63.2%–100% of mitochondrial genes in most plants have no introns [8, 9]. In addition, 30 introns were found in the other 15 *D. oleifera* mt genes; *nad1*, *nad2*, *nad5*, and *nad7* had 4 introns; *nad4*, 3 introns; and *cox2*, 2 introns.

The nucleotide composition of the whole mt genome (Table 3) was found to be A (27.27%), T (27.03%), C (22.90%), and G (22.80%). The overall GC content was 45.7%, consistent with that of other asterids (*V. macrocarpon* 45.33% [3], *D. carota* 45.41% [66], *Ilex pubescens* 45.55% [35], *Camellia sinensis* 45.70% [33], and *R. simsii* 45.86% [64]). The GC skew was positive in CDS regions and negative in the mitochondrial genome. Strikingly, the GC content of the PCGs (43.11%) was lower than that of other CDS regions (tRNAs and rRNAs).

**Table 3:** Composition and skewness of the *D. oleifera* mt genome.

*D.oleifera*	Size (bp)	A%	T%	G%	C%	A + T%	G + C%	AT-skew	GC-skew
Mitogenome	493958	27.27	27.03	22.8	22.9	54.3	45.7	0.004	-0.002
PCGs	32400	26.59	30.3	21.9	21.21	56.89	43.11	-0.065	0.016
tRNAs	2021	22.46	26.03	28.95	22.56	48.49	51.51	-0.073	0.124
rRNAs	5396	25.8	22.07	29.23	22.91	47.87	52.13	0.078	0.121

### 3.2 Repeat sequences analysis

Simple sequence repeats (SSRs, or microsatellites) are DNA stretches consisting of short, tandem units of sequence repetitions 1–6 base pairs in length [72]. We identified 87 SSRs in the *D. oleifera* mt genome. The proportions of different repeat units are shown in Table 1. Tetranucleotide repeats were the most abundant SSR type, constituting 68.97% of all identified SSRs, and there were 7 SSRs in di-, tri-, and pentanucleotide repeats, accounting for 8.05% of all identified SSRs. There were only three mono- and hexanucleotide repeats in the *D. oleifera* mt genome. AAAG/CTTT motifs (16) were most recurrent motifs, representing 18.39% of all identified SSRs (Table S1).

Tandem repeats (satellite DNA) are core repeating units of 1–200 bases repeated several times in tandem [73]. As shown in Table 4, 12 tandem repeats 6 to 30 bp long were observed in the *D. oleifera* mt genome.

**Table 4:** Distribution of perfect tandem repeats in the *D. oleifera* mt genome.

NO.	Size	Copy	Repeat sequence	Percent Matches	Start	End
1	30	1.9	TACTACAATCCGTACGATAACTAGAATCCG	82	123393	123450
2	18	2.2	GCTTGATTCGGTGTAAAC	90	143948	143987
3	20	2	TTTGATTTCATCTTCATATAC	90	176075	176115
4	14	2.8	GGAGCTGACACCCT	84	210479	210515
5	15	2.4	AAATAAAAAAATAAA	90	273479	273514
6	19	2.1	AACAACCTATCTTGCGACA	90	308468	308506
7	15	6.7	ACAACCTATTATGCG	70	308469	308572
8	18	2.1	AATACTAATAGAATAGAA	90	335217	335254
9	18	2.4	CATAGTCGCGAGCTGTTT	81	400200	400242
10	6	4.2	AAAGAA	100	409196	409220
11	18	5.2	TATTGATGATAGTGACGA	92	456597	456686
12	9	6.8	ATTGATGAT	73	456613	456673

**Figure 3:**
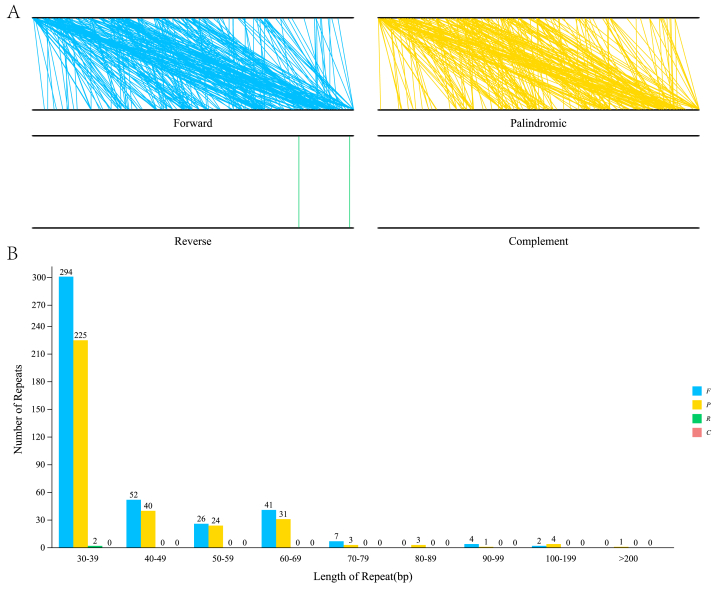
The repeats in the *D. oleifera* mt genome. A: The synteny between the mt genome and its copy showing the direct repeats. B: The length distribution of reverse and inverted repeats in the *D. oleifera* mt genome. The number on the histograms represents the repeat number of designated lengths shown on the horizontal axi.

In addition, 760 non-tandem repeats, with 30 bp or more in length, were detected in the *D. oleifera* mt genome. Of the 760 non-tandem repeats, 426 were direct, 332 were palindromic, and 2 were reverse. The longest direct-type repeat was 115 bp long, while the longest inverted repeat was 331 bp long (Table S2). As shown in Figure 3, the 30–39 bp repeats were most abundant for both repeat types.

The repetitive sequence in the *D. oleifera* mt genome was 31 kb, accounting for 6.33% of the total mitochondria. This is considered a medium proportion of repeats, higher than that in *Boea hygrometrica*, (1.5%) and *V. macrocarpon* (3%) and lower than that in *N. tabacum* (13%) [68] and *D. carota* (16%) [66]. The different proportions of repeats may be because the mitochondria of *B. hygrometrica*, *V. macrocarpon*, and *D. oleifera* are mainly short repeating units, whereas those of tobacco and carrots are mainly longer repeating units [66].

### 3.3 The prediction of RNA editing

The number of RNA-editing sites varies in different species and is usually frequent in angiosperm and gymnosperm mitochondria. We predicted 515 RNA-editing sites within all the 38 protein-coding genes (Table 5) in the *D. oleifera* mt genome, which is more than those in *A. thaliana* (441) [5], *Suaeda glauca* (261) [73], *Eucalyptus grandis* (470) [74], and *Citrullus lanatus* (463) [75] and less than those in gymnosperms with larger mt genomes, such as *Taxus cuspidata* (974), *Pinus taeda* (1179), *Cycas revoluta* (1206), and *Ginkgo biloba* (1306) [32]. However, whether the number of RNA-editing sites is positively correlated with the size of the mt genome requires further research.

**Table 5:** Prediction of RNA editing sites.

Type	Effect	Number	Percentage (%)
Hydrophilic	CGT (R) => TGT (C)	28	13.40
	CGC (R) => TGC (C)	13	
	CAT (H) => TAT (Y)	20	
	CAC (H) => TAC (Y)	8	
Hydrophobic	GCT (A) => GTT (V)	3	30.29
	GCG (A) => GTG (V)	7	
	GCC (A) => GTC (V)	2	
	CTT (L) => TTT (F)	13	
	CTC (L) => TTC (F)	5	
	CCT (P) => CTT (L)	19	
	CCG (P) => CTG (L)	35	
	CCC (P) => TTC (F)	6	
	CCC (P) => CTC (L)	7	
	CCA (P) => CTA (L)	45	
	CCT (P) => TTT (F)	14	
Hydrophilic-hydrophobic	TCT (S) => TTT (F)	44	47.57
	TCG (S) => TTG (L)	49	
	TCC (S) => TTC (F)	29	
	TCA (S) => TTA (L)	78	
	CGG (R) => TGG (W)	30	
	ACT (T) => ATT (I)	4	
	ACG (T) => ATG (M)	6	
	ACA (T) => ATA (I)	5	
Hydrophilic-stop	CGA (R) => TGA (X)	3	0.77
	CAA (Q) => TAA (X)	1	
Hydrophobic-hydrophilic	CCT (P) => TCT (S)	21	7.77
	CCC (P) => TCC (S)	9	
	CCA (P) => TCA (S)	6	
	CCG (P) => TCG (S)	4	

The selection of mitochondrial RNA-editing sites in *D. oleifera* shows a high degree of compositional bias. All RNA-editing sites are the C-T editing type, which is consistent with the fact that C-T is the most common editing type found in plant mt genomes [76, 77, 78]. In previous studies, almost half of the mitochondrial RNA editing occurred at the second codon position [73, 77]. The proportion of RNA-editing sites at the second codon position in the *D. oleifera* mt genome is also about 45.72% (235), slightly less than that at the first codon position (259; 50.39%). However, no editing site was found at the third position of triplet codons, consistent with the fact that RNA-editing sites are rare in plant mt genomes [73, 78].

Due to mitochondrial RNA editing, the *D. oleifera* mt genome has more RNA-editing sites but fewer editing types (Table 5). There were only 29 codon transfer types, corresponding to 14 amino acid transfer types, among the 515 RNA-editing sites. The types of transfer are comparable to those of most gymnosperms (30–40 codons; around 20 amino acids) [32, 76] but less than those of monocotyledonous and dicotyledonous plants (50–60 codons; around 30 amino acids) [74, 75, 78]. Among the 29 codon transfer types, TCA => TTA was the most common, with 78 sites. A leucine tendency after RNA editing, supported by the fact that 45.24% (233 sites) of the edits are converted to leucine, was found in the amino acids of predicted editing codons. After RNA editing, 43.59% of the amino acids remained hydrophobic. However, 47.57% of the amino acids were predicted to change from hydrophilic to hydrophobic, while 7.77% were predicted to change from hydrophobic to hydrophilic.

**Figure 4:**
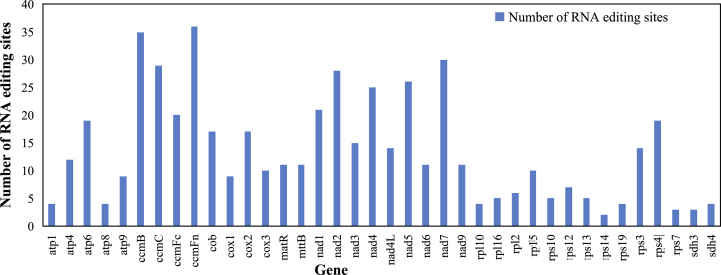
The distribution of RNA-editing sites in the *D. oleifera* mt protein-coding genes. The blue bars represent the number of RNA-editing sites of each gene.

The number and type of RNA-editing sites differed among the mt genomes of *D. oleifera* and other species. Like with most angiosperms [73, 76], ribosomal proteins (except *rps4*) and ATPase subunits (except *atp6*) had a relatively small number of RNA-editing-derived substitutions (1–12 sites), while the transcripts of NADH dehydrogenase subunits and cytochrome c biogenesis genes were significantly edited (11–36 sites; Figure 4), and *ccmFn* and *ccmB* had the most RNA-editing sites predicted (36, 35).

**Figure 5:**
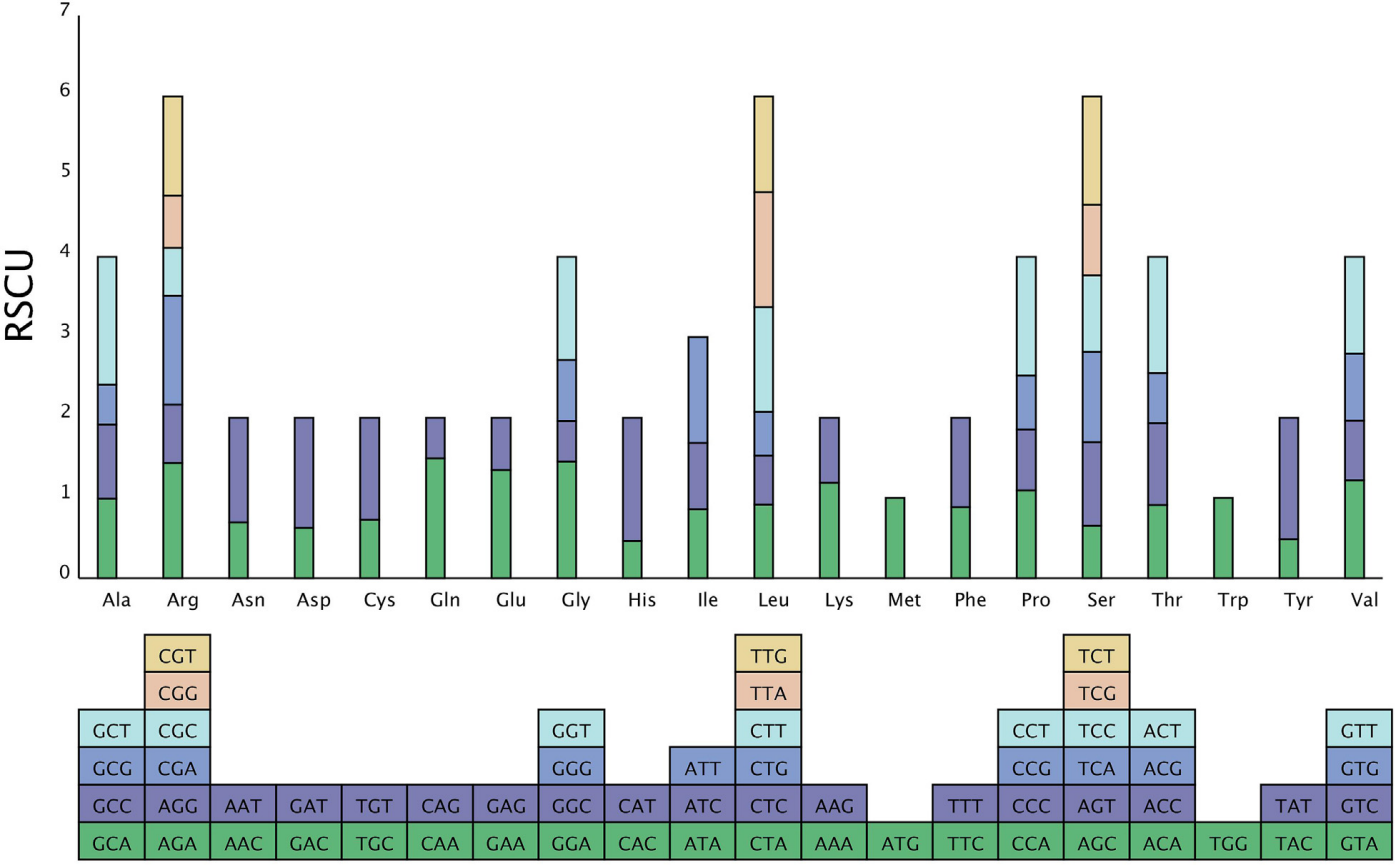
Relative synonymous codon usage in the *D. oleifera* mt genome.

In *D. oleifera,* 10,611 amino acids were encoded. The most frequently used amino acids were Leu (10.25%), Ser (9.23%), and Arg (6.86%), and the least common amino acids were Trp (1.52%) and Met (2.65%) (Figure 4). The relative synonymous codon usage (RSCU) value for *D. oleifera* for the third codon position is shown in Figure 5. Consistent with most of the currently studied mitochondrial genomes [1, 73, 76], the use of both two- and four-fold degenerate codons was biased toward the use of codons abundant in A or T.

### 3.4 Chloroplast-derived mitogenomic sequences

**Figure 6:**
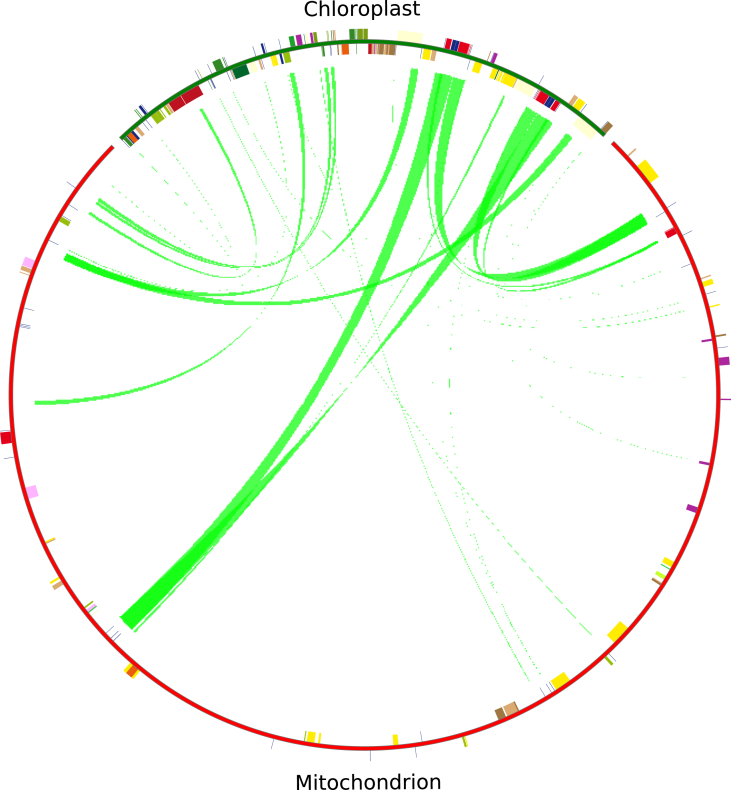
DNA and gene transfer between Chloroplast and Mitochondrial genomes in *D. oleifera*. The track shows complete genomes of cp and mt in green and red respectively.

The transfer of DNA sequences among chloroplast and mt genomes has been frequently observed in the mt genomes of plants [79]. In many cases, the chloroplast DNA content in the mt genomes of most plants is 3%–6%, sometimes reaching up to about 10% [80]. The *D. oleifera* mt genome contained 28 chloroplast insertions, ranging in length from 32 to 5703 bp (Figure 6, Table 6), with a total length of 32.83 kb, accounting for 6.65% of the total length of the genome, which is greater than the mitochondrial genome lengths of *Liriodendron tulipifera* (3%) [31], and *N. tabacum* (2.5%) [68]; comparable to those of *C. lanatus* (6%) [75], *E. grandis* (6%) [74], and *Oryza sativa* (6.3%) [81]; and less than those of *Vitis vinifera* (8.8%) [36] and *Cucurbita pepo* (11.5%) [75].

**Table 6:** Chloroplast insertions in the mitochondrial genome of *D. oleifera*.

Chloroplast insertion	Start	End	Length	Chloroplast genes carried	Mitochondrial gene
1	102039	107741	5703	*rps12-rrn16-rrn23-trnA-UGC-trnI-GAU-trnV-GAC*	*nad5/trnA-TGC/trnI-GAT/trnV-GAC/*
2	137054	142756	5703	*rps12-rrn16-rrn23-trnA-UGC-trnI-GAU-trnVGAC*	*nad5/trnA-TGC/trnI-GAT/trnV-GAC*
3	132935	136943	4009	*rps12-rrn23-rrn4.5-rrn5-trnN-GUU-trnR-ACG*	*trnN-GTT/trnR-ACG/*
4	107852	111860	4009	*rps12rrn23-rrn4.5-rrn5-trnN-GUU-trnR-ACG*	*trnN-GTT/trnR-ACG*
5	148183	150621	2439	*ycf15-ycf2*	ORF
6	94174	96612	2439	*rps12-ycf15-ycf2*	ORF
7	55481	56973	1493	*atpB-atpE*	ORF
8	66698	67774	1127	*psbE-psbF-psbJ-psbL*	ORF
9	24713	25651	939	*rpoB-rpoC1*	ORF
10	68737	69666	939	*petG-petL-trnPUGG-trnWCCA*	*trnW-CCA*
11	103614	104477	888	*rps12-rrn16*	*rrn18*
12	140318	141181	888	*rps12-rrn16*	*rrn18*
13	124702	125419	719	*rps12-ndhA-ndhH*	*nad5*
14	65137	65375	245	*petA*	*nad1*
15	31892	32085	197	*trnDGUC*	*nad1/trnD-GTC*
16	47055	47223	171	*trnSGGA*	*trnS-GGA*
17	36672	36818	147	*psbC*	*nad1*
18	1096	1190	96	*psbA*	ORF
19	9282	9370	92	*trnSGCU*	*trnS-GGA*
20	133140	133220	82	*rps12-trnN-GUU*	*nad1/trnN-GTT*
21	111575	111655	82	*rps12-trnN-GUU*	*nad1/trnN-GTT*
22	54808	54886	79	*TrnM-CAU*	*nad1/trnM-CAT*
23	155661	155735	77	*TrnI-CAU*	*nad1/ccmC/orf*
24	89060	89134	77	*rps12-trnI-CAU*	*nad1/ccmC/orf*
25	95837	95897	61	*rps12-ycf2*	*nad1/orf*
26	148898	148958	61	*ycf2*	*nad1/orf*
27	155661	155692	32	*trnICAU*	ORF
28	89103	89134	32	*rps12-trnICAU*	ORF

Among the transfer DNA sequences, some chloroplast protein-coding genes, such as *atpB*, *atpE*, *rps12*, *rpoB*, *petA*, *psaA*, and *psbC*, lost their integrity while migrating from the cp to the mitochondria, and only partial sequences of those cp-derived PCGs could be found in the *D. oleifera* mt genome (Table 6). In the *D. oleifera* mt genome, 11 chloroplast-derived tRNAs with a complete sequence were identified: *trnA-UGC*, *trnD-GUC*, *trnI-GAU*, *trnM-CAU*, *trnN-GUU*, *trnP-UGG*, *trnR-ACG*, *trnS-GCU*, *trnS-GGA*, *trnV-GAC*, and *trnW-CCA*. The different completeness levels of the transferred PCGs and tRNA genes showed that tRNA genes are much more conserved in the mt genome than PCGs, indicating that tRNA genes play an indispensable role in mitochondria. The transfer of these tRNAs can be traced back to the retention of an earlier horizontal gene transfer event. In accordance with the present results, cp-derived *trnM-CAU* first appeared in gymnosperms [82]; cp-derived *trnD-GUC* mainly appeared in dicotyledons, not in monocotyledons [76]; and cp-derived *trnM-CAU* and *trnD-GUC* were both found in the *D. oleifera* mt genome. However, the absence of cp-derived *trnH-GTG*, which is commonly found in angiosperms [3, 74, 76, 82], and the presence of cp-derived *trnA-UGC*, lost during early evolution of terrestrial plants [80, 83], indicate that special evolutionary events may be occurring during *D. oleifera* formation.

### 3.5 Phylogenetic, Ka/Ks and gene arrangement analysis

**Figure 7:**
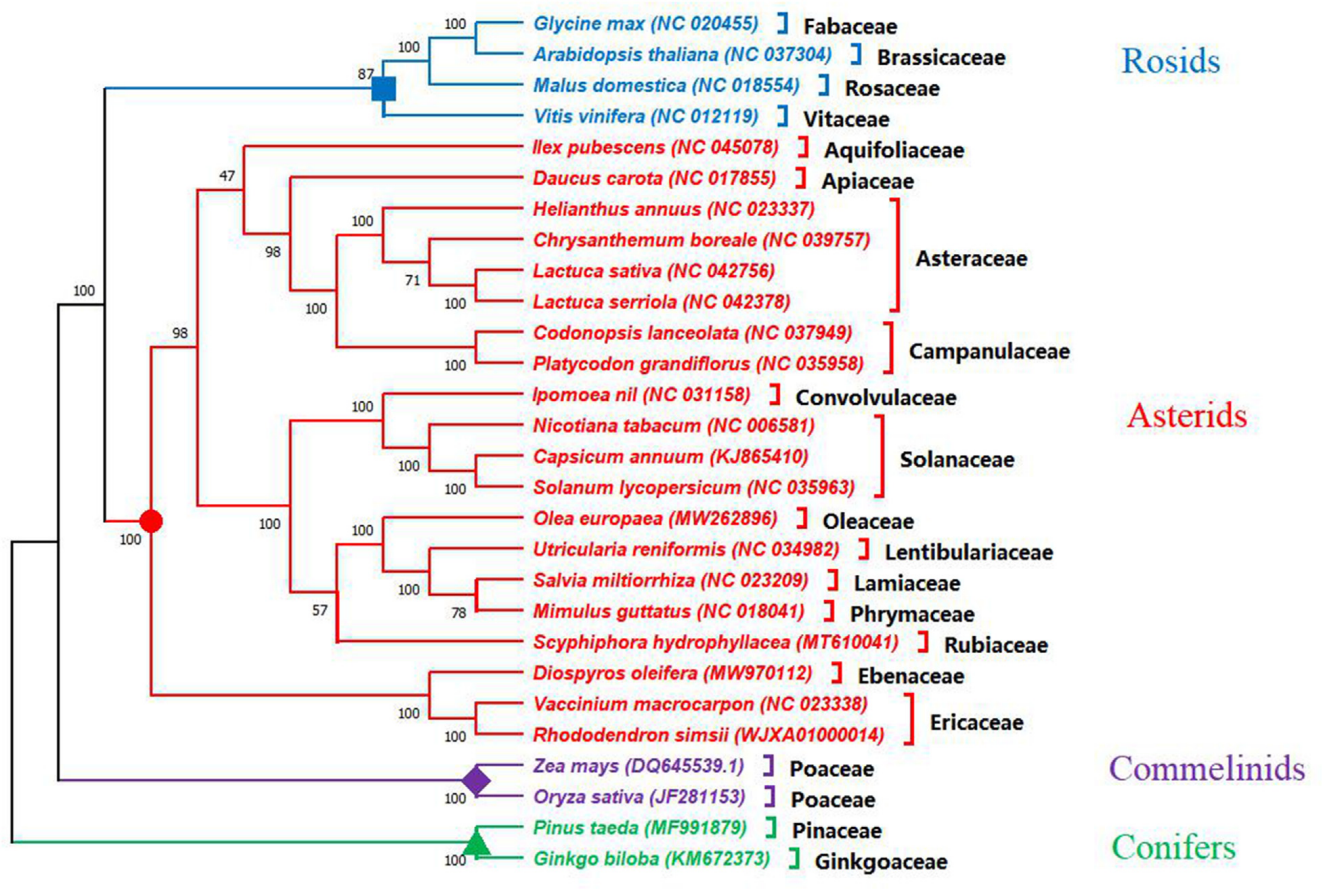
The phylogenetic relationships of *D. oleifera* with other 27 plant species using the maximum likelihood (ML) analysis. The bootstrapping values are listed in each node. The number after the species name is the GenBank accession number. Colors indicate the groups that the specific species belongs.

To detect the evolutionary status of the *D. oleifera* mt genome, a phylogenetic analysis was performed on *D. oleifera*, together with 27 other species: 23 eudicots (19 asterids and 4 rosids), 2 monocotyledons, and 2 gymnosperms (designated as outgroups). Phylogenetic relationships were analyzed using the concatenated dataset (28 PCGs: *atp1*, *atp4*, *atp6*, *atp8*, *atp9*, *ccmB*, *ccmC*, *ccmFC*, *ccmFN*, *cob*, *cox1*, *cox2*, *cox3*, *matR*, *nad1*, *nad2*, *nad3*, *nad4*, *nad4L*, *nad5*, *nad6*, *nad7*, *nad9*, *rpl5*, *rps12*, *rps13*, *rps3* and *rps4*) through ML phylogenetic analysis. The abbreviations and accession numbers of the mt genomes investigated in this study are listed in Table S1. As outgroups, the two gymnosperms were distinct from the other angiosperms. The phylogenetic tree (Figure 7) strongly supported the separation of asterids from rosids and the separation of eudicots from monocots. Moreover, the taxa from 20 families (Apiaceae, Aquifoliaceae, Asteraceae, Brassicaceae, Campanulaceae, Convolvulaceae, Ericaceae, Ebenaceae, Fabaceae, Ginkgoaceae, Lamiaceae, Lentibulariaceae, Oleaceae, Phrymaceae, Pinaceae, Poaceae, Rosaceae, Rubiaceae, Solanaceae, and Vitaceae) were well clustered. In addition, the monophyly of *D. oleifera*, which belongs to the single genus of *Diospyros* in the Ebenaceae family, was well supported based on mt genomes (Figure 7). Consistent with previous comparative genome studies [23, 28, 29], this study also found that the clade united *V. macrocarpon* and *R. simsii* and then formed a sister cluster with the Ebenaceae family with high confidence (bootstrap value of 100%). In general, the phylogenetic tree topology was in line with the evolutionary relationships among those species, indicating the consistency of traditional taxonomy with the molecular classification.

To evaluate selective pressures during the evolutionary dynamics of protein-coding genes among closely related species, the nonsynonymous (Ka) and synonymous (Ks) substitution ratio (Ka/Ks) was calculated. For the Ka/Ks calculation, 28 PCGs from the *D. oleifera* mt genome were compared with the mt genomes of 27 species.

**Figure 8:**
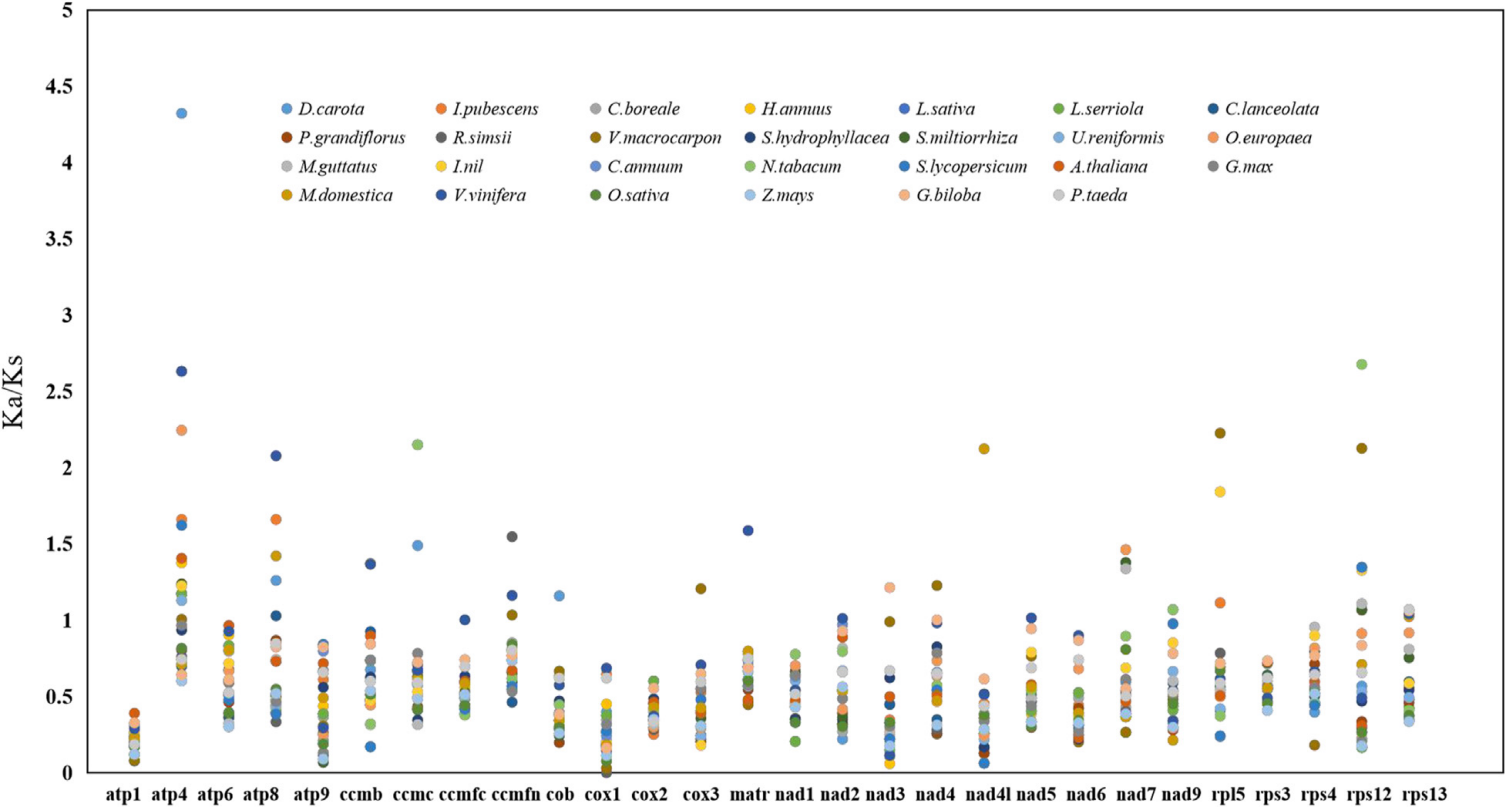
The Ka/Ks values of 28 protein-coding genes of *D. oleifera* versus 27 species.

As shown in Figure 8, for the gene-specific substitution rates, Ka/Ks ranged from 0.031 at the *cox1* gene in *V. macrocarpon* to 4.321 at the atp4 gene in *D. carota*. In 58 cases (except *Glycine max*, *O. sativa*, *Platycodon grandiflorus*, *Scyphiphora hydrophyllacea* and *Z. mays*), the Ka/Ks values of *D. oleifera* gene-specific substitution rates were higher than 1, compared with 22 other species, suggesting positive selection during evolution. Among the 22 species, nine substitution genes with higher Ka/Ks values were found between the *D. oleifera* and *V. vinifera* mt genomes and six genes between the *D. oleifera* and *V. macrocarpon* mt genomes. The *atp4* and *atp8* genes exhibited the highest average rate (1.348 and 0.751) and 15 and 5 Ka/Ks values above 1, respectively, suggested to be the result of positive or relaxed selection [2]. However, most genes had undergone negative selection pressures during evolution, supported by the fact that the Ka/Ks values of 654 proteins, accounting for 91.59% of the proteins in *D. oleifera*, were less than 1 compared to the other plant species. The *atp1* and *cox1* genes have the smallest average Ka/Ks values (0.212 and 0.272), indicating strong purifying selection [34, 84]. These results show that mt genes are highly conserved during the evolutionary process in green plants.

**Figure 9:**
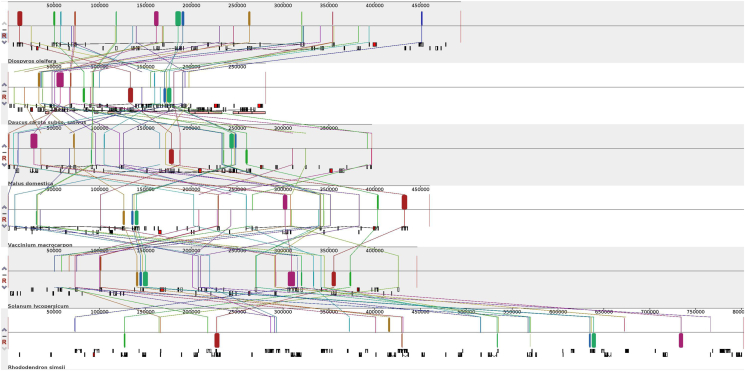
Synteny analysis of *D. oleifera* and other five species mitogenomes as generated with Mauve. The sizes and relative positions of the homologous fragments varied across mitogenomes.

Because of no mt genome of any species in Ebenaceae has been reported, Synteny of entire mitochondrial genomes was only compared among four Asterids (including three Ericales, one Apiales, and one Solanales) and one Rosids species in this study to assess the degree of structural rearrangement between different lineages. Figure 9 and Figs.1 showed that the rearrangement of mitochondrial genes has been widely occur among these six species, which is accords with many mitogenome observations [20, 37, 38, 39, 41, 42]. When using *D. oleifera* as a reference genome, The dot-plot analyses showed sequences or synteny were seldom shared, and only short stretches of synteny among species (Figs.2). These Large rearrangement events have indicated differentiation within these six species mitogenome. Understandably, species that have close evolutionary relationships share more clusters [20, 41, 42], for example, In general, longer synteny sequences with higher similarity were found between *D. oleifera* and *V. macrocarpon* than that between *D. oleifera* and *M. domestica.*

Among genus *Diospyros*, The most well-known species is *D. kaki*, which has been cultivated as an important fruit crop, due to its edible fruit [25]. However, *D. kaki* are hexaploid (2n = 6× = 90) or nonaploid (2n = 9× = 135) and their origin, and polyploidization mechanisms are unclear, which has hampered genome sequencing and molecular breeding [23, 28]. Phylogenetic analyses based on the nuclear [23, 28] and chloroplast [26, 27] genome and mtDNA non-coding fragments [85] have indicated that *D. oleifera* is more closely related to *D. kaki* [24] and could be used as a model plant for studies of *Diospyros* [24, 26]. So, as the nuclear and mt genome of hexaploid cultivated persimmon both remains unpublished, the availability of the *D. oleifera* mt genome provides more alternative comparable reference for *D. kaki* than *D. lotus* does. In addition, our results will lay the foundation for identifying further evolutionary relationships within Ebenaceae. However, due to the lack of adequate representative mitogenomes, more *Ebenaceae mitogenomes* are needed to be sequenced to better resolve the phylogeny and evolutionary biology of Ebenaceae.

## 4 Conclusions

Here, we presented the first mitochondrial genome assembly and annotation of an Ebenaceae model plant *Diospyros oleifera* as well as the mitochondrial genome in the family Ebenaceae. The mitogenome was 493,958 bp in length, contained 39 protein-coding genes, 27 transfer RNA genes, and 3 ribosomal RNA genes. Comparative analysis of gene structure, codon usage, repeat regions and RNA-editing sites shows that *rps2* and *rps11* genes are missing, and a clear bias of RNA-editing sites is existing in the *D. oleifera* mt genome. In addition, the phenomenon that intracellular tRNA genes transferred frequently from chloroplasts to mitochondria was also observed in *D. oleifera*. Moreover, Phylogenetic analysis based on the mt genomes of *D. oleifera* and 27 other taxa indicates consistency in molecular and taxonomic classification. Furthermore, The Ka/Ks analysis based on code substitution revealed that most of the coding genes had undergone negative selections, indicating the conservation of mt genes during the evolution. These results will help in better understanding the features of the *D. oleifera* mitochondrial genome and lay the foundation for identifying further evolutionary relationships within Ebenaceae. However, due to the lack of adequate representative mitogenomes, more *Ebenaceae mitogenomes* are needed to be sequenced to better resolve the phylogeny and evolutionary biology of Ebenaceae.

## Declarations

### Author contribution statement

Yang Xu: Conceived and designed the experiments; Performed the experiments; Analyzed and interpreted the data; Contributed reagents, materials, analysis tools or data; Wrote the paper.

Yi Dong: Performed the experiments.

Wenqiang Cheng; Haidong Gao, Lei Liu and Lei Xu: Analyzed and interpreted the data.

Kaiyun Wu: Contributed reagents, materials, analysis tools or data.

Bangchu Gong: Conceived and designed the experiments; Contributed reagents, materials, analysis tools or data; Wrote the paper.

### Funding statement

Ph.D. Yang Xu was supported by National Key R & D Program of China [2018YFD1000606].

Ph.D. Yang Xu was supported by National Key R & D Program of China [2019YFD1000600].

Bangchu Gong was supported by Key Project for New Agricultural Cultivar Breeding in Zhejiang Province, China [2021C02066-10].

### Data availability statement

Data associated with this study [The final annotated mt genome sequences of *D. oleifera*] has been deposited at NCBI GenBank under the accession number MW970112.

### Declaration of interests statement

The authors declare no conflict of interest.

### Additional information

No additional information is available for this paper.

## Appendix A Supplementary data

Fig.S1 Analysis of conservative gene clusters between the *D. oleifera* mt genome and other plant mt genomes. Fig.S1

Fig.S2 Dot-plot graphs indicating regions of synteny between mitochondrial genomes compared to *D. oleifera* as the reference. Fig.S2

Table S1 Distribution to different repeat type classes in the *D. oleifera* mt genome. Table S1

Table S2 Frequency of classified SSR motifs in the *D. oleifera* mt genome. Table S2

Table S3 Repeats (≥30bp) in the *D. oleifera* mt genome. Table S3
