# Supplementary figures and images for: Characterization and phylogenetic analysis of the complete mitochondrial genome sequence of Diospyros oleifera, the first representative from the family Ebenaceae
Source: Heliyon. 2022 Jul 6;8(7):e09870. doi: 10.1016/j.heliyon.2022.e09870 (PMC9283892; doi:10.1016/j.heliyon.2022.e09870)

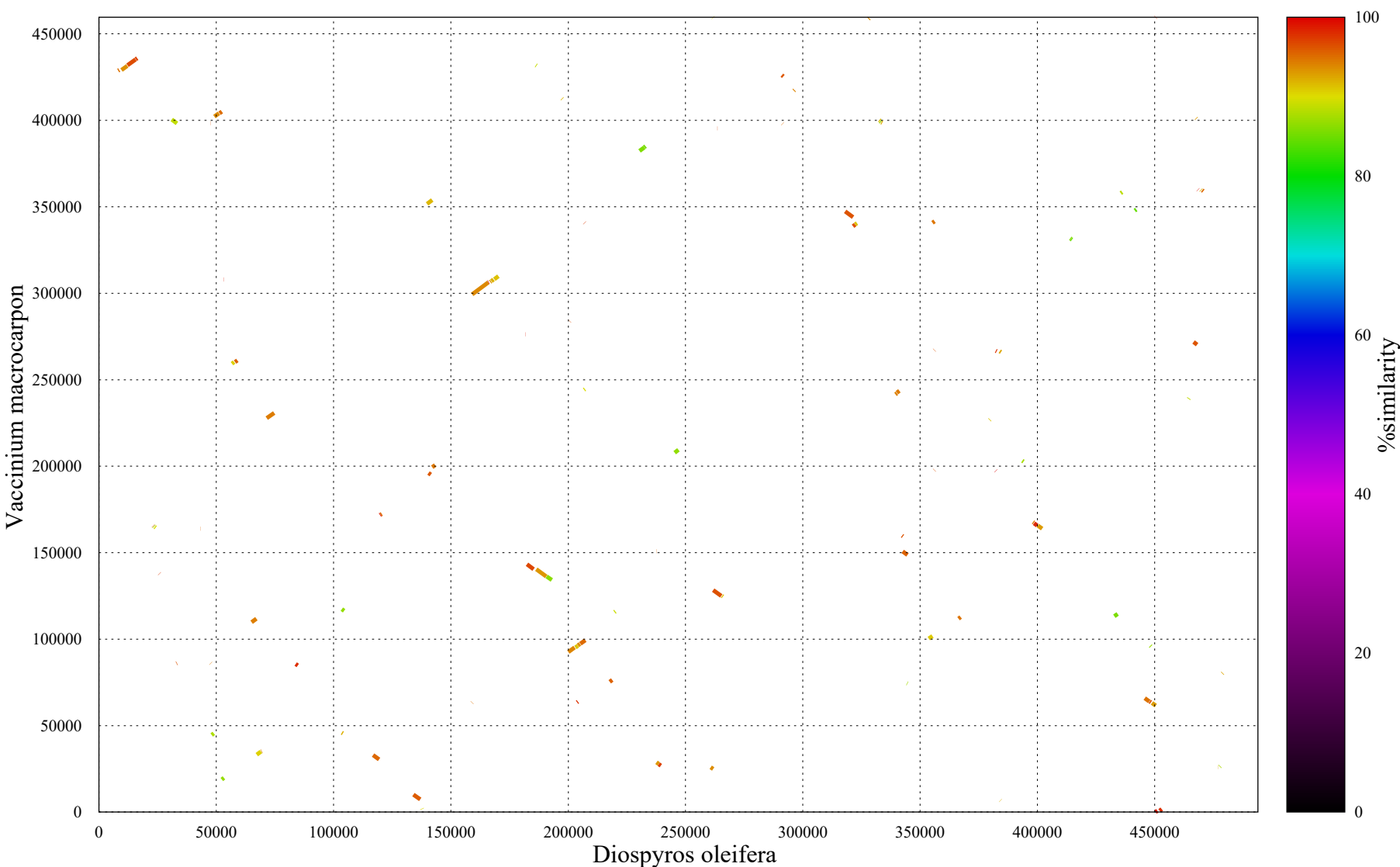

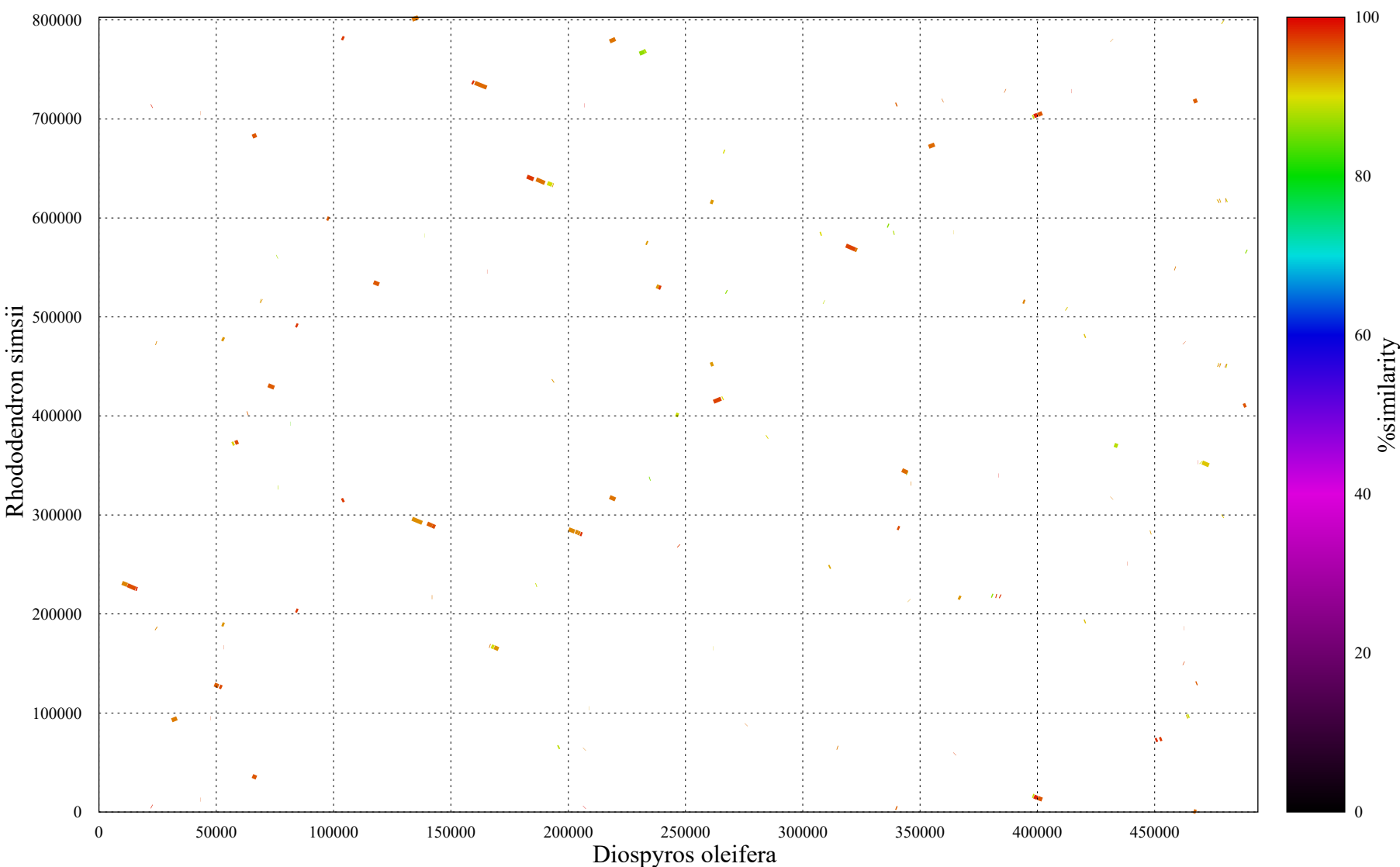

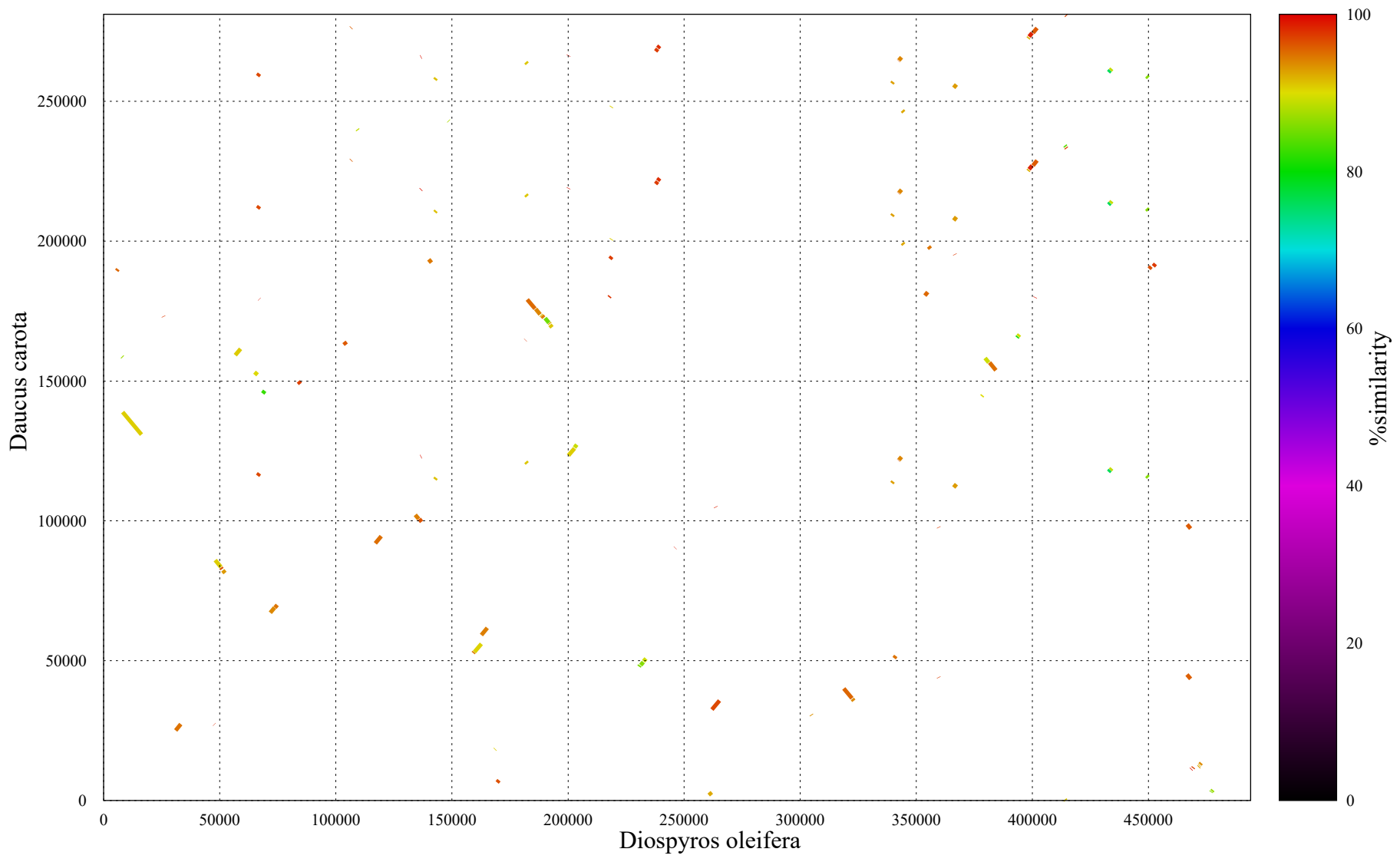

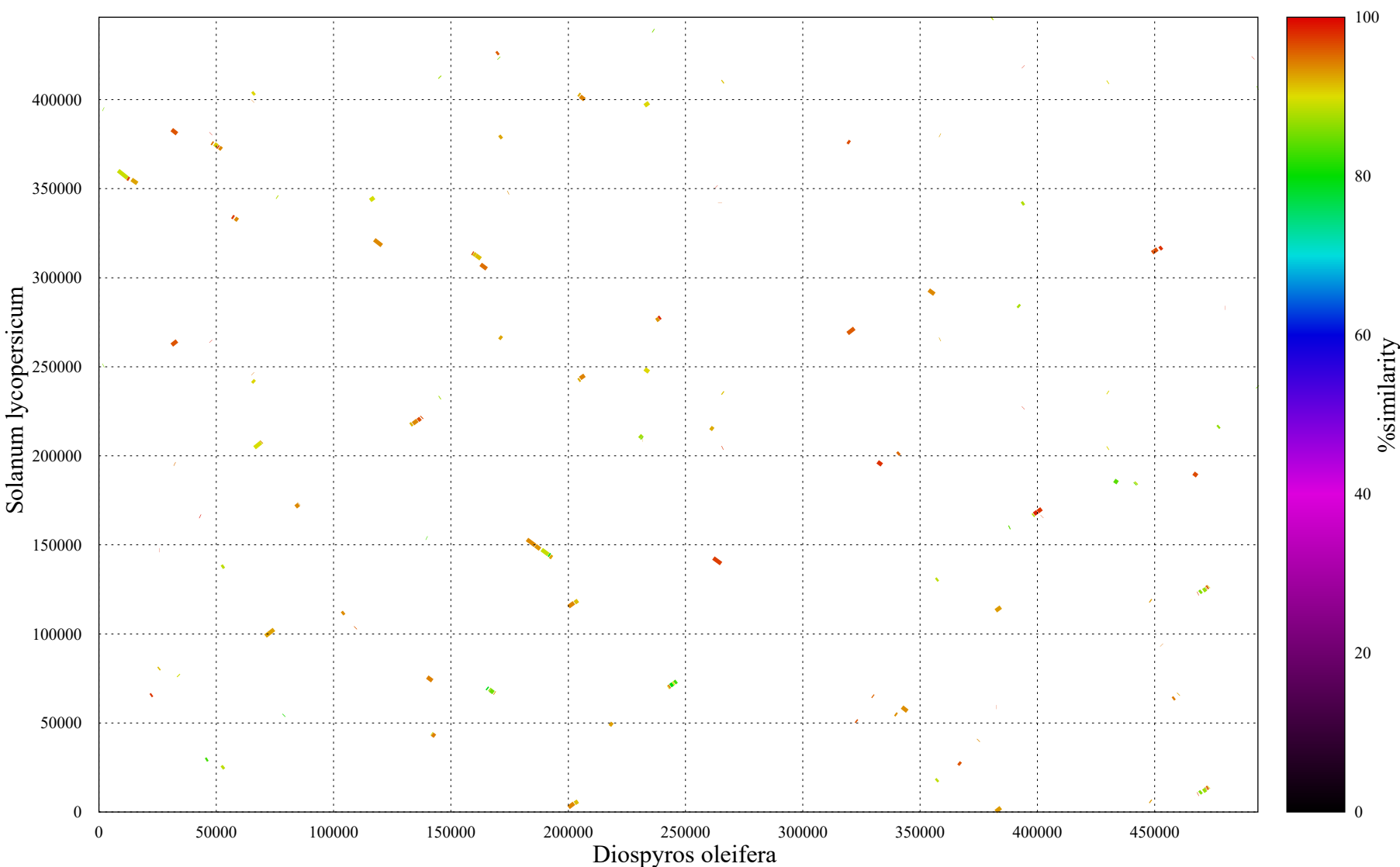

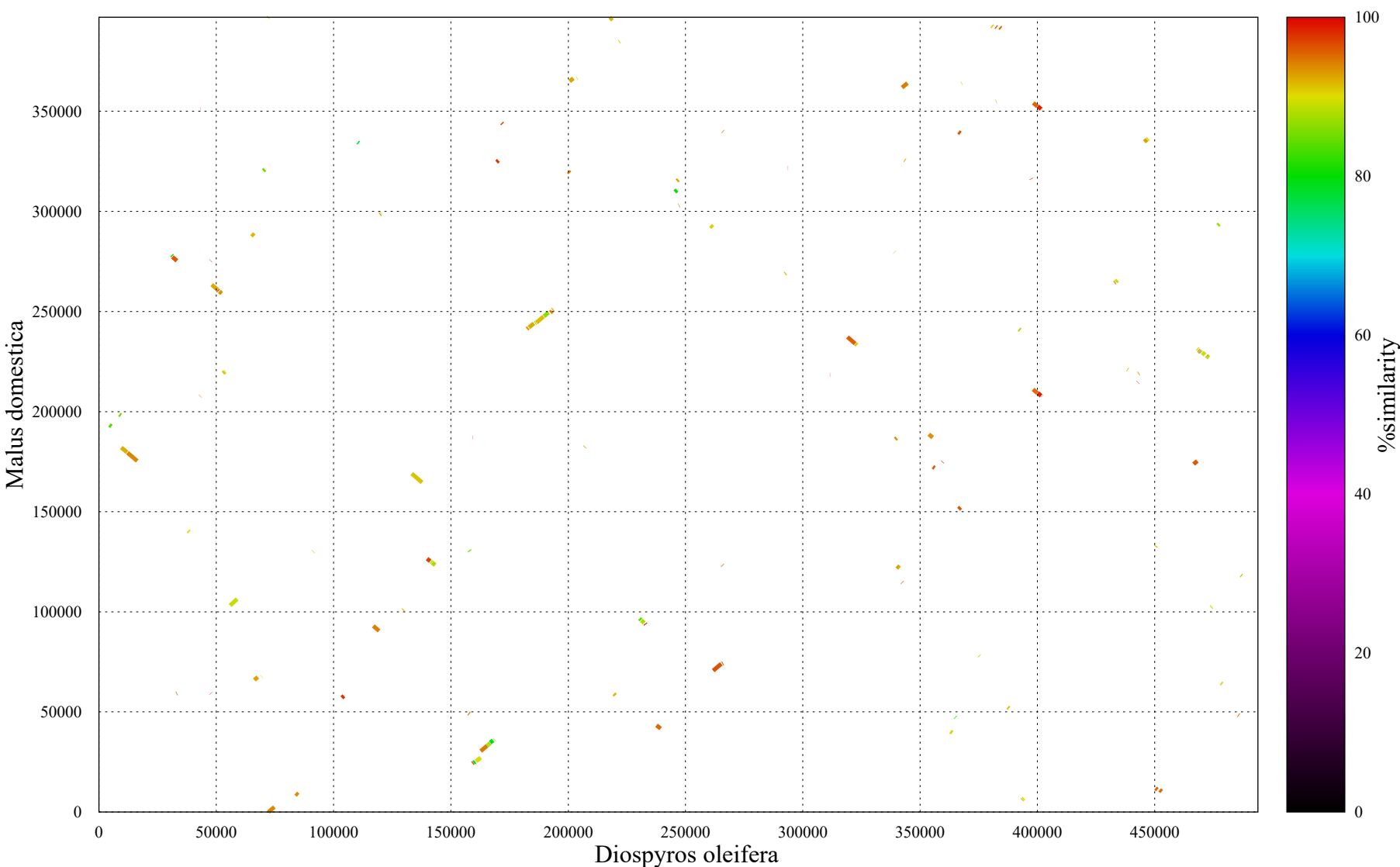

Supplement: Fig.S1 — Analysis of conservative gene clusters between the D. oleifera mt genome and other plant mt genomes. [file mmc1.pdf]

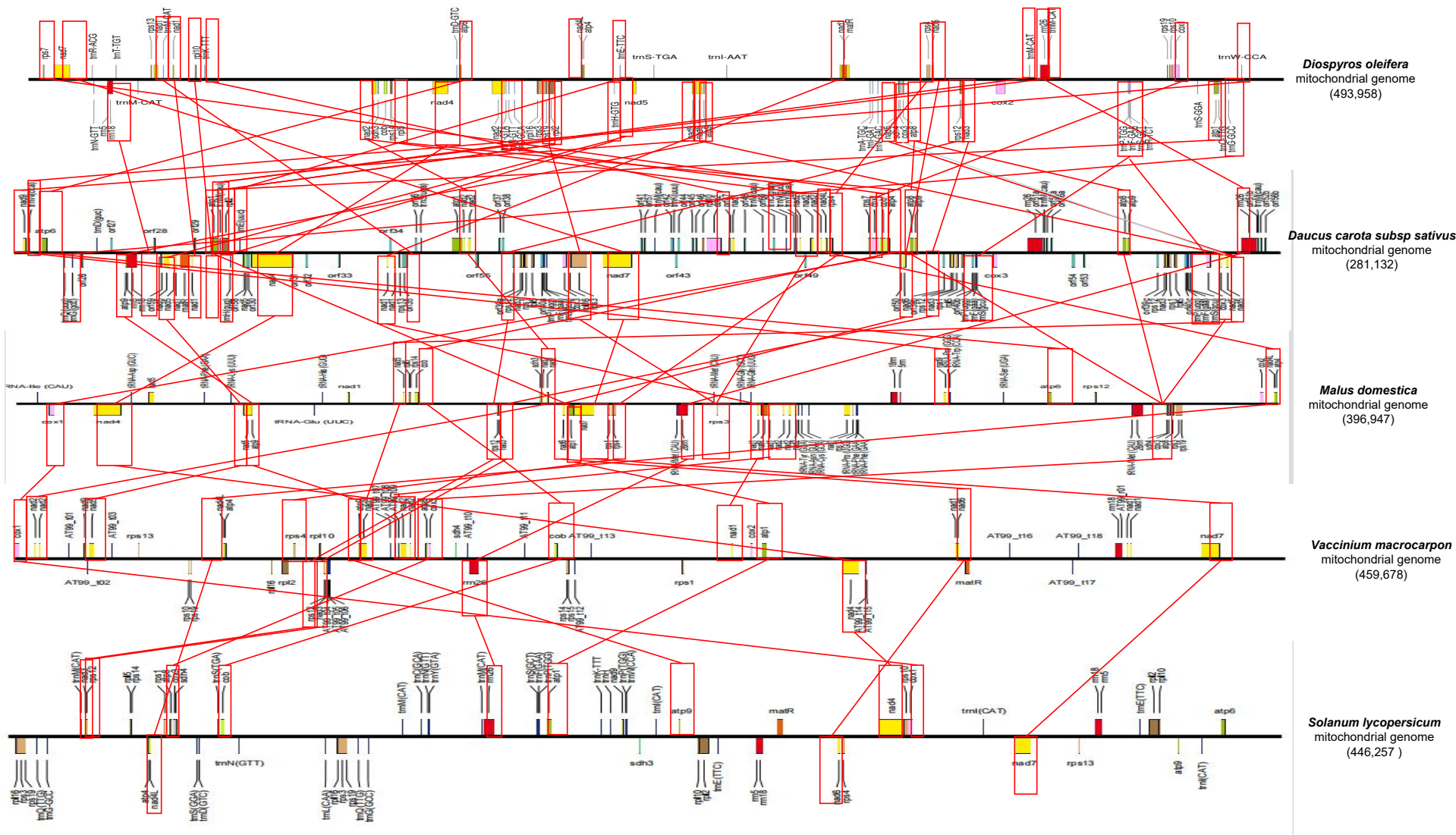

Supplement: Fig.S2 — Dot-plot graphs indicating regions of synteny between mitochondrial genomes compared to D. oleifera as the reference. [file mmc2.pdf]
